# Alkyl‐Linked Porphyrin Porous Polymers for Gas Capture and Precious Metal Adsorption

**DOI:** 10.1002/smsc.202000078

**Published:** 2021-05-05

**Authors:** Yeongran Hong, Vepa Rozyyev, Cafer T. Yavuz

**Affiliations:** ^1^ Department of Chemical and Biomolecular Engineering Korea Advanced Institute of Science and Technology (KAIST) 291 Daehak-ro, Yuseong-gu Daejeon 34141 Republic of Korea; ^2^ Graduate School of EEWS KAIST 291 Daehak-ro, Yuseong-gu Daejeon 34141 Republic of Korea; ^3^ Pritzker School of Molecular Engineering The University of Chicago 5640 South Ellis Avenue Chicago IL 60637 USA; ^4^ Advanced Membranes and Porous Materials Center (AMPM) Division of Physical Sciences and Engineering (PSE) King Abdullah University of Science and Technology (KAUST) 5640 South Ellis Avenue Thuwal 23955-6900 Saudi Arabia

**Keywords:** CO_2_ storage, e-waste recycling, Friedel–Crafts polymerization, gold reduction, platinum recovery, water treatment

## Abstract

In gas adsorption and metal recovery, inexpensive and covalently bonded porous polymers offer industrial feasibility, despite the challenge of having reactive functionalities while maintaining porosity. Herein, three highly porous covalent organic polymers (COPs), COP‐210, COP‐211, and COP‐212, with porphyrin functionalities that are readily synthesized by a Friedel–Crafts reaction using chlorinated solvents as linkers are reported. The polymers exhibit competitive adsorption capacities for CO_2_, H_2_, and CH_4_. Their porphyrin sites proved particularly effective in precious metal recovery, where COPs exhibit high selectivity toward gold, platinum, palladium, and silver. Analysis reveals that reductive metal capture is prevalent for gold and silver. Platinum is also captured through a combination of reduction and chelation. The gold adsorption capacities are 0.901–1.250 g g^−1^ with fast adsorption kinetics at low pH. COP‐212 selectively recovers 95.6% of gold from actual electronic waste (e‐waste) collected from junkyards. The results show that the inexpensive and scalable porous porphyrin polymers offer great potential in gas capture, separation, and precious metal recovery.

## Graphical abstract

Electronic waste (e‐waste) contains valuable metals that can pay for the cost of recycling computers, cellphones, and electronics. Herein, porphyrin porous polymers that capture precious metals, particularly gold, from among 30 coordinating metals that are commonly found in e‐waste are introduced. These polymers are stable in strong acids and bases, and scalable in kilogram quantities using affordable starting materials.

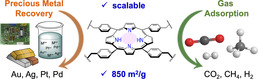

## 1 Introduction

Porous polymers have attracted much attention during the past decades due to their diverse functionalities, controllable pore structures, and easy preparation. With these features, porous polymers have been studied in a variety of applications such as gas separation, metal adsorption, water purification, catalysis, photocatalysis, and energy storage.^[^ ^1^, ^2^, ^3^, ^4^, ^5^, ^6^ ^]^ Porphyrins are easily accessible and unique building blocks for the construction of functional and scalable porous networks but used rather infrequently. The porphyrin ring structure is constituted by four pyrroles and can provide a strong adsorption site for both gas molecules and metal ions.^[^ ^7^, ^8^, ^9^, ^10^, ^11^, ^12^ ^]^

In gas separation applications, the porphyrin pyrrolic nitrogens offer enhanced binding for CO_2_.^[^ ^9^, ^13^, ^14^, ^15^ ^]^ Recent studies that focus on this feature include porphyrin‐based conjugated microporous polymers (CMPs) with azide groups,^[^ ^16^ ^]^ a porphyrin‐ and pyrene‐based CMP (Por–Py–CMP),^[^ ^14^ ^]^ a triazine‐functionalized porphyrin‐based porous organic polymer (TPOP‐1),^[^ ^17^ ^]^ and 3D Mn^II^‐porphyrin metal–organic framework (MOF).^[^ ^18^ ^]^ Porphyrin‐based porous structures also show ability to store hydrogen (H_2_) gas.^[^ ^13^, ^19^, ^20^ ^]^ Polyporphyrins with high surface areas (e.g., over 1500 m^2^ g^−1^) and functionalized with thiophenyl groups showed 5% mass increase after H_2_ adsorption at 77 K and 65 bar.^[^ ^20^ ^]^ But, the less than stellar performances reveal the need to provide better and economical accessibility^[^ ^21^ ^]^ to the complex functionality of porphyrins.

Porous polymers, in general, are also emerging as selective adsorbents for metals from wastewater.^[^ ^22^, ^23^, ^24^, ^25^ ^]^ Precious metals such as gold, platinum, palladium, and silver are promising target metals for recovery because of their high values and widespread use in chemical industries.^[^ ^26^, ^27^ ^]^ These metals can be recovered from electronic waste (e‐waste), which is called urban mining.^[^ ^28^, ^29^ ^]^ According to the forecasts, the amount of e‐waste will reach 74 million tons a year in 2030 and the precious metals will be even more important since high‐tech products increasingly need these metals.^[^ ^30^, ^31^, ^32^ ^]^ Some nonporous adsorbents were developed for precious metals, particularly with high gold adsorption capacities, but mostly, their selectivities were tested using only a few metals and were unsatisfactory on other requisites such as adsorption kinetics and desorption readiness.^[^ ^33^, ^34^, ^35^, ^36^ ^]^ This is not enough for real e‐waste applications, and therefore, more proper adsorbents should be developed. Porous polymers with tailored functionalities and large contact areas are expected to solve these problems. In our previous study, amidoxime‐functionalized polymer, also known as being active to uranium ions,^[^ ^37^, ^38^ ^]^ was studied for the gold recovery.^[^ ^22^ ^]^ The nitrile groups in the polymer can be modified to amidoxime groups without losing the porosity of the polymer, so it was possible to compare the functionality effects on the metal adsorption. It was found that the amidoxime groups are more effective adsorption sites for the gold than nitrile groups but less so when compared to chelating substrates such as porphyrins. Porous polymers with porphyrin building blocks only recently used for precious metal capture by our group,^[^ ^39^ ^]^ but the two‐step synthesis and low yields in polymer making led us to explore more convenient and industrially feasible versions.

In this article, we report inexpensive and scalable porphyrin‐based covalent organic polymers (COPs) for precious metal capture and gas separations. The porphyrin units were linked by a rapid one‐pot Friedel–Crafts (FC) reaction with three different linkers, dichloromethane (DCM), chloroform (CHCl_3_), and 1,2‐dichloroethane (DCE), to give three porous polymers of COP‐210, COP‐211, and COP‐212, respectively. The polymers have high surface areas and are predominantly microporous. The polymers showed high adsorption selectivity toward precious metals such as gold, platinum, palladium, and silver in a mixed metal test using an acidic solution of 30 different metals. With the combined features of microporosity and porphyrin functionality, and the inexpensive preparations, the selective gas and metal capture by the developed polymers were found to promise industrial feasibility.

## 2 Results and Discussion

### 2.1 Preparation of COP‐210, COP‐211, and COP‐212

**Figure 1:**
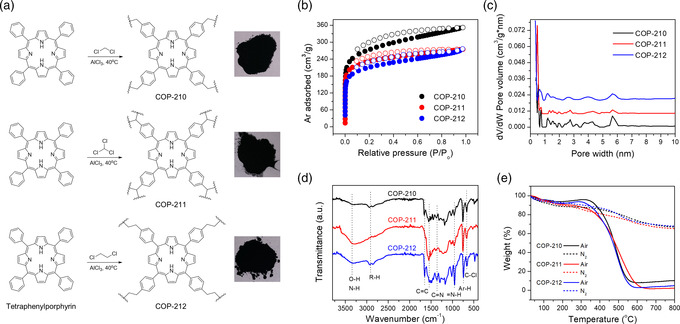
Synthesis of alkyl‐linked porphyrin‐based COPs. a) Synthetic schemes and the photos of the products, b) argon adsorption and desorption isotherms at 87 K, c) pore size distribution using NLDFT calculations, d) FT–IR spectra, and e) TGA results under air and nitrogen atmosphere for COP‐210, COP‐211, and COP‐212.

FC alkylation is one of the most promising synthetic methodologies for making highly porous networks with C–C bonded frameworks because of the availability of the precursors and reagents.^[^ ^40^ ^]^ Here, we made three different porphyrin‐based COPs using FC alkylation polymerization. The synthesis followed our previous solvent‐linked FC polymerization method.^[^ ^41^ ^]^ There, we use a commercially available monomer, meso‐tetraphenylporphyrin, aluminum chloride (AlCl_3_), and chlorinated solvents to prepare inexpensive polymers. The synthesis proceeds in a single step under mild reaction conditions (40 °C) and open to air. Chlorinated solvents, DCM, CHCl_3_, and DCE serve as both linkers and solvents to give three different polymers indexed as COP‐210, COP‐211, and COP‐212, respectively (**Figure** **1**). Due to the highly reactive benzylchloride‐like intermediate formations and strong AlCl_3_ Lewis acid, reaction progress is driven to polymer formation. The porous polymers were obtained with at least 80–90% yields. The products are insoluble in common solvents and stable under air and moisture, providing easy workup and purifications.

Porosity of porphyrin‐based COPs was analyzed from argon adsorption and desorption isotherms at 87 K. Materials showed high Brunauer–Emmett–Teller (BET) surface areas (685–856 m^2^ g^−1^) with predominantly microporous morphology (84–92%). Among these COPs, DCM‐linked COP‐210 showed the highest surface area of 856 m^2^ g^−1^ (**Table** **1**). Nonlocal density functional theory (NLDFT) pore size distributions using a slit pore model confirm predominant microporous structure of the materials (Figure 1). Chloroform‐linked COP‐211 featured the highest microporosity, 92% of the total pore volume. We believe that higher microporosity was formed as a result of higher cross‐linking of three dentate linker CHCl_3_ compared to two dentate linkers (DCM and DCE). DCE‐linked polymer displayed both the lowest surface area (685 m^2^ g^−1^) and microporous nature (84%) among the three structures, which can be explained by flexible nature of the dimethylene linkers that does not have rigid backbone for strained network formation. These findings on the new porous polymers are consistent with our previous study.^[^ ^41^ ^]^ Morphology of COPs was analyzed using scanning electron microscopy (SEM) and the results were shown in Figure S7, Supporting Information. SEM images revealed that grains of COP‐210 are mainly flat structures, COP‐212's are spherical, but COP‐211 varied from flat to spherical particles (Figure S7, Supporting Information). The COP‐212 showed much smaller particle size (≈100 nm) than COP‐211 (≈5 μm) and COP‐210 (≈25 μm). This indicates that although most of the surface area of COP‐210 and COP‐211 are coming from their intrinsic porosity, COP‐212 shows significant interparticle void space, which inadvertently contributes to the porosity. Fourier‐transform infrared spectroscopy (FT–IR) analysis of COPs demonstrates characteristic porphyrin bands at 1365 cm^−1^ (C=N) and 954 cm^−1^ (N—H), aromatic bands at 1500–1650 cm^−1^ and 740 cm^−1^ (Ar–H), and aliphatic linker bands at 2700–3000 cm^−1^, which illustrates the successful formation of porphyrin‐based network polymers. In addition, the presence of C–Cl stretching at 665 cm^−1^ indicates the dangling unreacted linker from solvents. Thermogravimetric analysis (TGA) of these COPs under nitrogen and air shows thermal stability up to 300 °C. The desorption of adsorbed water and solvents (up to about 150 °C and ≈20% of mass decrease) also confirms high porosity of the materials (Figure 1).

**Table 1:** Porosimetry and gas adsorption properties of COP‐210, COP‐211, and COP‐212

Sample	SA_BET_ [m^2^ g^−1^]	*V*_total_ [cm^3^ g^−1^]	*V*_micro_ [cm^3^ g^−1^]	CO_2_ uptake 273 K, 1.1 bar [mmol g^−1^]	CO_2_ uptake 298 K, 1.1 bar [mmol g^−1^]	CH_4_ uptake 273 K, 1.1 bar [mmol g^−1^]	CH_4_ uptake 298 K, 1.1 bar [mmol g^−1^]	H_2_ uptake 77 K, 1.1 bar [mmol g^−1^]
COP‐210	856	0.445	0.376	4.30	2.85	1.33	0.74	8.88
COP‐211	769	0.368	0.341	3.47	2.21	1.44	0.75	8.24
COP‐212	685	0.324	0.279	3.01	1.86	1.30	0.66	7.11

Elemental composition of COPs was measured by combustion (CHNS) analysis and results are shown in Table S1, Supporting Information. Carbon to nitrogen ratio from elemental analysis indicates that single‐carbon‐linked COP‐210 and COP‐211 have six extra carbon (50:4) per one tetraphenylporphyrin monomer. Since DCE has two carbons per molecule, DCE‐linked COP‐212 has 13.7 extra carbon (57.7) per tetraphenylporphyrin monomer. Higher hydrogen to nitrogen ratios of COP‐212 also support this finding. In addition, all COPs show elemental content other than carbon, nitrogen, and hydrogen. This is due to the residual AlCl_3_ catalyst, unreacted dangling alkyl chlorides, and its hydrolyzed forms. This is supported by the presence of C–Cl and O–H stretchings in FT–IR measurements and leftover mass (Al_2_O_3_) after air TGA measurement. Despite washing with concentrated HCl solution and Soxhlet washing with methanol, COPs contain residual aluminum. Leftover mass (Al_2_O_3_) in air TGA reveals 4.3% aluminum in COP‐210 as compared to 0.9% in COP‐211 and 1.5% in COP‐212. This might be due to the higher surface area of COP‐210. Inductively coupled plasma mass spectrometry (ICP–MS) measurements show that residual Al is leached after precious metal adsorption experiments, suggesting it is replaced by strongly binding metal ions (Figure S6, Supporting Information).

### 2.2 Gas Uptake of COPs

**Figure 2:**
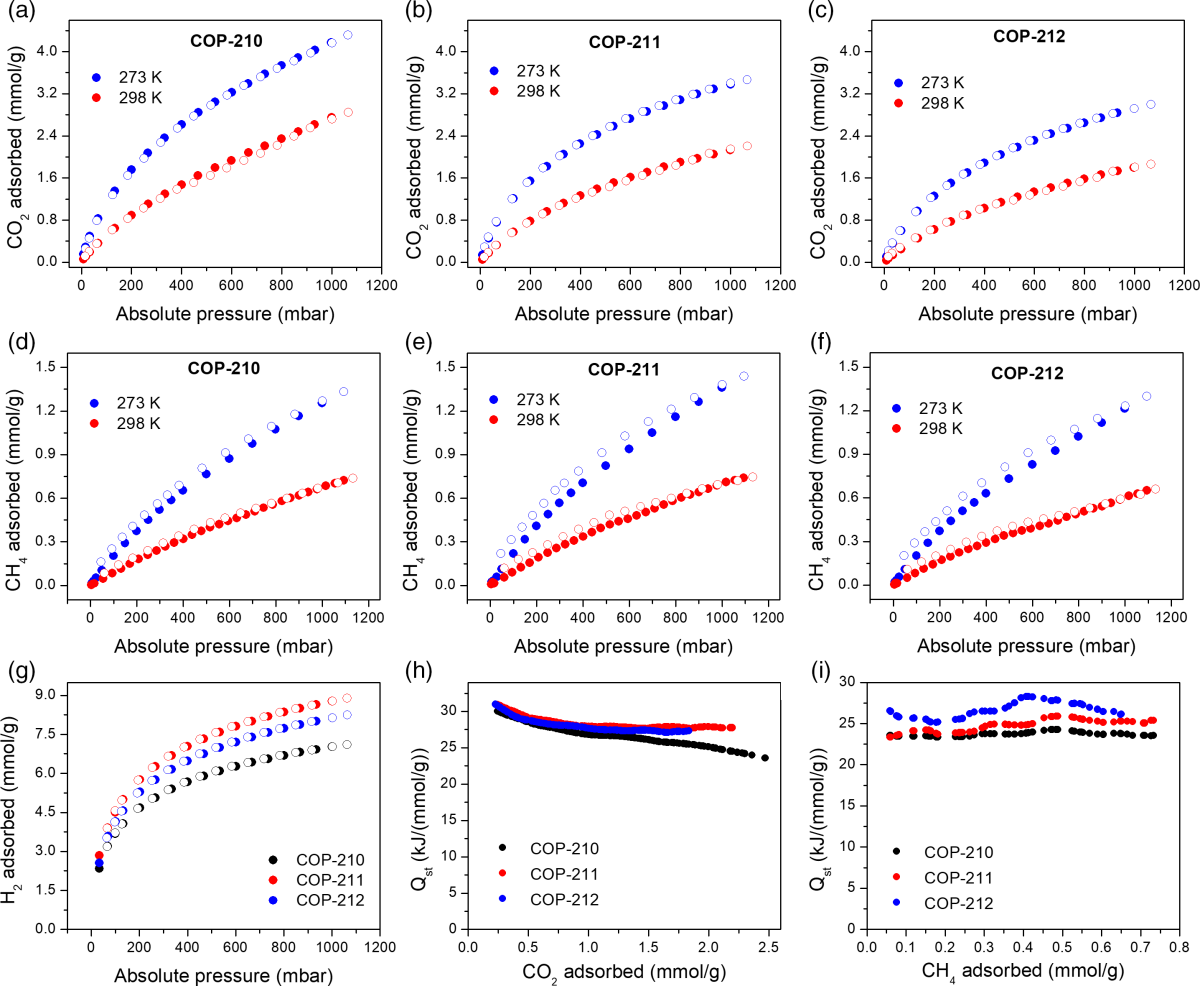
Gas uptakes of COPs. Carbon dioxide uptake of a) COP‐210, b) COP‐211, c) COP‐212 at 273 K and 298 K. Methane uptake of d) COP‐210, e) COP‐211, f) COP‐212 at 273 K and 298 K. g) Hydrogen uptake of COP‐210, COP‐211, and COP‐212 at 77 K. *Q*_st_ calculations for h) CO_2_ and i) CH_4_ uptake of the COPs.

To bring out the gas separation features of these porous polymers, we studied the carbon dioxide (CO_2_), methane (CH_4_), and hydrogen (H_2_) adsorption properties. CO_2_ adsorption isotherms revealed no hysteresis indicating mainly physisorptive binding, with capacities ranging from 3.01 to 4.30 mmol g^−1^ at 273 K and 1.1 bar (Table 1 and **Figure** **2**). CO_2_ adsorption enthalpy (*Q*_st_) was calculated from adsorption isotherms at 273 and 298 K. Since these COPs possess similar CO_2_‐philic porphyrin chemistry, they showed similar *Q*_st_ values (30–31 kJ mol^−1^). Therefore, their CO_2_ uptake capacities exhibited direct correlation with total BET surface areas; the higher the surface area the higher CO_2_ capacities. These values are comparable or better than the nitrogen‐rich and highly porous structures such as BILP‐6 (See Table S6, Supporting Information, for comparison table).^[^ ^42^, ^43^, ^44^ ^]^

H_2_ adsorption isotherms measured at 77 K featured similar trend as CO_2_ adsorption. Adsorption capacities at 1.1 bar is linearly correlated by BET surface areas of materials. Capacities varied from 7.11 mmol g^−1^ (COP‐212) to 8.88 mmol g^−1^ (COP‐210), which are among the leading materials in H_2_ adsorption (See Table S6, Supporting Information, for comparison table). For example, COP‐210 stores more H_2_ than higher surface area materials like COF‐102 (3530 m^2^ g^−1^, 5.96 mmol g^−1^).^[^ ^45^ ^]^ Considering the scalable and inexpensive production, these COPs are among the promising materials in hydrogen storage.

Similarly, the porous polymers exhibited good performance in CH_4_ adsorption, with capacities up to 1.44 mmol g^−1^ (COP‐210) at 273 K and 1.1 bar. We studied pressures up to slightly above 1 bar to offset the possibility of swelling behavior that we previously reported for alkyl‐linked porous polymers.^[^ ^41^ ^]^ When the CH_4_ *Q*_st_ values were calculated, DCE‐linked COP‐212 had the higher binding energy (*Q*_st_ = 27 kJ mol^−1^) than DCM‐linked COP‐210 and chloroform‐linked COP‐211 (*Q*_st_ = 24 kJ mol^−1^). This is due to the methane‐philic dimethylene framework, which has been previously reported.^[^ ^41^ ^]^ As a result, despite having lower surface area and micropore volume, COP‐212 showed similar methane adsorption as COP‐210. Surprisingly, despite having relatively lower surface area and lower *Q*_st_ value, COP‐211 had the highest CH_4_ uptake capacity, possibly due to the highly cross‐linked tridentate linker. It should be noted that CH_4_ has larger kinetic diameter (0.38 nm) than CO_2_ (0.33 nm) and H_2_ (0.29 nm). Therefore, CH_4_ adsorption might be more sensitive to pore sizes than CO_2_ and H_2_.

### 2.3 Metal Capture Studies

In metal capture, metal binding selectivity is one of the (if not the most) important properties of adsorbents along with adsorption capacity, kinetics, and recyclability, since solutions almost always include competing metals. To investigate the metal selectivity of the new COPs, we previously developed a rapid testing method^[^ ^39^ ^]^ by using commercial standard solutions that contain common elements and precious metals (Figure S1, Supporting Information). COP‐210, COP‐211, and COP‐212 showed high adsorption efficiencies for precious metals such as gold (99%), platinum (94–96%), and palladium (99%) in the standard‐1 solution. In the mixed standard solution‐1 and 2, gold (99%), platinum (96–99%), and palladium (99%) were still well captured and also, copper (10–30%) exhibited high adsorption efficiencies. The high adsorption of silver (51–66%) and copper (10–30%) was observed in the standard solution‐2. Therefore, gold, platinum, palladium, silver, and copper were selected for the further adsorption studies using stock solutions.

The COPs were treated with the single metal solutions of gold, platinum, palladium, silver, and copper at a high concentration of 3000 ppm. The adsorbents were treated with the spiked metal solutions to derive maximum capacities. The adsorbed metal amounts were then measured by ICP–MS (Table S1, Supporting Information). Gold recorded the highest adsorption of around 30 wt%. Over 8 wt% of platinum, palladium, and silver were loaded on COPs. These high metal loadings demonstrated the effective single metal adsorption of the developed COPs for these metals. The COPs also captured copper, but as seen in the selectivity test results, the adsorption was less effective, resulting in the adsorption amounts of 1.3–2.9 wt%.

**Figure 3:**
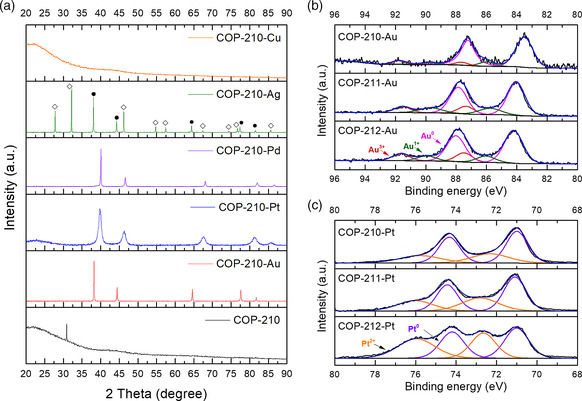
Spectroscopic analysis of the metal‐loaded COPs. a) Powder XRD patterns of copper, silver, palladium, platinum, and gold‐loaded COP‐210 and nonmetalated COP‐210, b) XPS spectrum of gold (4f) of gold‐loaded COPs, and c) XPS spectrum of platinum (4f) of platinum‐loaded COPs. In the XRD pattern of COP‐210–Ag, the symbols of diamond (◊) and filled circle (•) indicate silver chloride and silver nanoparticles, respectively. XRD patterns of metal‐loaded COP‐211 and COP‐212 can be found in Figure S2, Supporting Information.

Upon analysis through powder X‐ray diffraction (XRD), we observed reductive uptake; one possible reason for the high selectivity of the preferred metals. The XRD patterns of metal‐loaded COPs clearly displayed the formation of nanoparticles of gold, platinum, palladium, and silver (**Figure** **3**a). In the silver‐loaded COPs, silver chloride was also found. This is because there are remaining chlorides in the adsorbent due to the use of aluminum chloride in the polymer synthesis. The sizes of metal nanoparticles from Scherrer equation were ≈11.1–12.2 nm, 9.8–11.8 nm, 11.1–11.3 nm, and 24.3–36.3 nm for gold, platinum, palladium, and silver‐loaded COPs, respectively. However, bigger gold particles in several microns were also observed in transmission electron microscopy (TEM) images (Figure S4, Supporting Information).

The X‐ray photoelectron spectroscopy (XPS) data also supported the metal reduction mechanism (Figure 3b,c). The XPS spectrum of COP‐210–Au revealed three oxidation states of gold as 0 at 83.53 (4f_7/2_) and 87.23 (4f_5/2_) eV, +1 at 85.98 and 89.78 eV, and +3 at 87.68 and 91.78 eV, meaning that +3 states of gold ions were reduced to +1 and 0 states. The adsorbent also reduced the platinum ions, which have a little lower reduction potential than gold ions (AuCl_4_^−^ + 3e^−^ → Au + 4Cl^−^, +1.002 V, [PtCl_4_]^2−^ + 2e^−^ → Pt + 4Cl^−^, +0.755 V), showing two oxidation states as 0 at 70.98 (4f_7/2_) and 74.33 (4f_5/2_) and +2 at 72.33 and 75.78 eV in the XPS spectrum of COP‐210–Pt.^[^ ^46^, ^47^ ^]^ As mentioned, the reduction potentials of precious metals such as gold, platinum, palladium ([PdCl_4_]^2−^ + 2e^−^ → Pd + 4Cl^−^, +0.591 V), and silver (Ag^+^ + e^−^ → Ag, +0.7996 V) are higher than those of other common metals, resulting in the higher adsorption efficiencies to these metals in the metal selectivity tests. Therefore, we conclude that the developed COPs can adsorb these metals first through chelation but then drive further through the reduction mechanism. The metals also can be adsorbed by chemically binding with the polymer structures, as +3 and +2 states of gold ions and +2 state of platinum ions were found in the XPS study, although it is not clear which functional groups these metals were interacted with.

Copper uptake is interesting since copper is not a metal with high reduction potential (Cu^2+^ + 2e^−^ → Cu, +0.3419 V) such as the aforementioned precious metals, but it was captured by the COPs with moderate affinities in the metal selectivity test. In the XRD pattern of copper‐loaded COPs, the peaks of metallic copper particles did not appear, although the adsorbents include 1.3–2.9% of copper when treated with high concentration of copper solutions. It was expected that the copper ions were bound by the polymer structures, especially at the porphyrin sites. To confirm this, a porphyrin solution was prepared and reacted with gold, platinum, palladium, copper, iron, cobalt, nickel, and zinc solutions. Water soluble porphyrin, 5,10,15,20‐tetra(4‐pyridyl)‐21H,23H‐porphyrin, was used because of the extremely low solubility of meso‐tetraphenylporphyrin in water. The porphyrin solution was mixed with the metal solutions, and after stirring 24 h, the light absorption of the mixtures in ultraviolet and visible (UV/vis) ranges was measured. The color change was observed only in the copper and porphyrin solutions, where the porphyrin peak at 446 nm was shifted to 427 nm only when the copper and porphyrin mixed. In other metals and porphyrin solutions, the peak shift was not found. The peak shift in the copper and porphyrin mixture implies that the copper ions were bound by the porphyrin rings to form metalloporphyrin. Iron, cobalt, nickel, and zinc were selected for this test because their atomic numbers are close to that of copper and have similar ionic sizes. However, unlike copper, these metals did not display interactions with porphyrins, as no shifts were observed in their UV/vis absorption spectra. Gold ions were even reduced when reacted with porphyrin monomers, so the UV/vis absorption changes in the mixture of porphyrin and gold could not be observed (Figure S5, Supporting Information). The study on how copper has stronger affinity to the porphyrin ring is of interest to us and we are studying it further.

### 2.4 Gold Adsorption and Desorption of COPs

**Figure 4:**
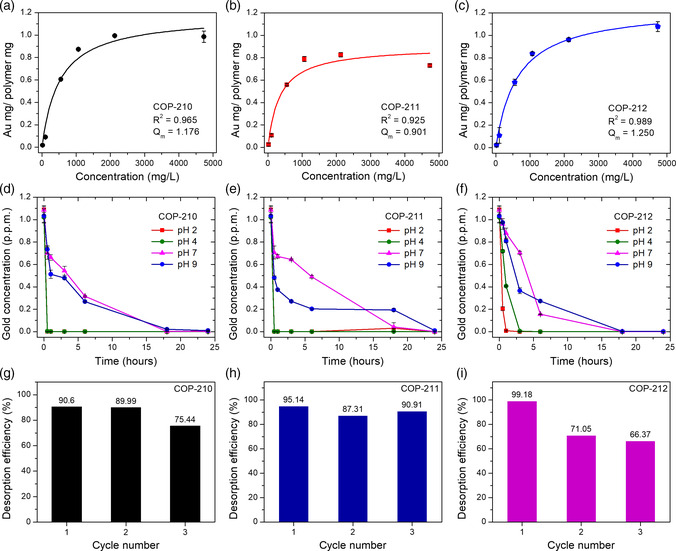
Gold adsorption and desorption of COPs. Gold adsorption isotherms of a) COP‐210, b) COP‐211, and c) COP‐212. Time‐dependent gold concentration changes of d) COP‐210, e) COP‐211, and f) COP‐212 at varying pH. Desorption efficiencies at three repetitive adsorption–desorption processes of g) COP‐210, h) COP‐211, and i) COP‐212.

The gold adsorption capacities were found to be 1.176, 0.901, and 1.250 g g^−1^ for COP‐210, COP‐211, and COP‐212 (**Figure** **4**a–c), respectively. Theoretical gold adsorption amounts can be calculated based on the nitrogen percent from the elemental analysis results and those values were 0.241, 0.222, and 0.210 g g^−1^ for COP‐210, COP‐211, and COP‐212, respectively. Compared to the theoretical gold adsorption amounts, the observed gold adsorption amounts were much higher, which implies that the gold reduction process is mainly involved for the gold capturing. Very low values of Langmuir constant (*K*_L_) mean the weak affinity between adsorbents and adsorbates, which also supports the gold reduction mechanism (Table S3, Supporting Information). These adsorption capacities were higher than many other reported gold adsorbents (Table S5, Supporting Information). The effective gold adsorption by the developed COPs was observed at varying pH of the gold solution. As a result, it was revealed that the COPs captured gold ions at lower pH more effectively, as adsorbing more than 99% of gold ions within 30 min, whereas basic pH adversely affected the gold capture. This is probably because of the gold ion speciation from auryl chloride ions to hydroxide complexes as increasing pH, and the anionic repulsion can happen between gold hydroxides and deprotonated porphyrin units in the polymer structures. This repulsion also prevents reductive capture since adsorption would not take place. Three polymers showed similar trends in their gold adsorptions at tested pH ranges of 2–9 (Figure 4d–f).

For the efficient recovery of precious metals, the adsorbed metals ought to be separated from the adsorbents. The metals can exist as nanoparticles, ionic forms bound to porphyrins, or both. In any case, we suspected that strong acids and chelating reagents such as thiourea can be helpful for their desorption. The desorption conditions such as desorption solution, temperature, and desorption time should be as mild as possible so that the adsorbents can be regenerated and reused for the next cycle. Thiourea is a good replacement of cyanide which is a highly toxic reagent that is normally used for gold leaching in gold mining and recovery industries. Therefore, for the metal desorption, the mixture of dilute strong acids (HCl and HNO_3_) and thiourea was used and the solutions were heated to 40 °C. In these conditions, gold adsorbed on the polymers was effectively recovered with 87–99% of desorption efficiencies. After the third cycle, structures were confirmed by comparing the before and after FT–IR spectra, although some minor changes were observed compared to the spectra of pristine COPs (Figure S3, Supporting Information). As the cycles proceeded, the desorption efficiencies were decreased due to the remaining gold in the polymer structures. In contrast, the desorption efficiencies of platinum, palladium, and copper were 20–35%. As explained, these metals exist as particles and ionic forms and it seemed that metal ions are strongly bound to the polymer structures and not easily desorbed. Almost all gold was reduced to form particle in various sizes, so they can be more easily dissolved by the desorption reagents. Similarly, silver was more readily retrieved by using the HNO_3_ and thiourea mixture (Table S2a, Supporting Information). When the gold adsorption and desorption processes were repeated, the desorption efficiencies decreased during the three consecutive cycles. This was due to the trapped gold in the polymers after the desorption processes. The gold adsorption amounts increased as the cycles progressed, which means that the polymers did not lose the gold adsorption properties during the cycles (Figure 4g–i and Table S2b, Supporting Information).

### 2.5 Application of COP‐212 in Real e‐Waste Solutions

**Figure 5:**
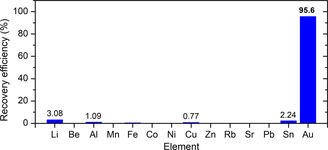
Gold recovery from actual e‐waste solution using COP‐212. The porous polymer shows selective gold capture from an actual e‐waste digest, where transition metal cations are present.

Finally, COP‐212 was tested for the gold recovery from an actual e‐waste since it showed the highest gold adsorption capacity among the three porous polymers. To make a treatment solution, metals on collected printed circuit boards (PCBs) were dissolved by using strong acids. The COP‐212 was added to the prepared e‐waste solution and tumbled for 24 h. Even though the gold concentration is much lower than other metals, COP‐212 successfully captured gold with near quantitative adsorption efficiency of 95.6% (**Figure** **5** and Table S4, Supporting Information). This result indicates that the developed COPs can be applied in gold recovery from metal leachate of e‐waste.

## 3 Conclusion

Three new porphyrin‐based porous polymers were synthesized using a one‐step easily scalable synthesis. Commercial tetraphenylporphyrin was reacted separately with three reagents, DCM, CHCl_3_, and DCE, which acted as both linkers and solvents. The porphyrin units and the reagents were linked with C—C bonds through the Friedel–Crafts reaction. The developed polymers were highly porous (685–856 m^2^ g^−1^ SA_BET_) and predominantly microporous. They were studied for the gas and metal adsorption applications. In the gas uptake tests, they showed increasing CO_2_ uptakes (from 3.01 to 4.30 mmol g^−1^ at 273 K and 1.1 bar) in line with the increasing surface areas due to the physisorptive adsorptive contributions. Similarly, the polymers adsorbed more H_2_ (from 7.11 to 8.88 mmol g^−1^) with higher surface areas. For the CH_4_ uptake, COP‐211 with lower surface areas and *Q*_st_ value possessed the highest CH_4_ uptake capacity possibly due to the highly cross‐linked tridentate linker. Meanwhile, the COPs can be utilized for the precious metal recovery. It was found that the polymers adsorbed precious metals such as gold, platinum, palladium, silver, and copper more selectively than other common metals. With 1.176, 0.901, and 1.250 g g^−1^ of gold adsorption capacities, the polymers exhibited fast gold adsorption kinetics at low pH. The adsorbed gold was readily desorbed by the mild desorption conditions and the adsorption properties of the polymers were maintained to three repetitive adsorption and desorption processes. The COPs displayed industrially viable gas and metal adsorption performances and as they are scalable and inexpensive, proving that these polymers are promising materials in both applications. As an example, COP‐212 was applied in the metal leachate from an actual e‐waste. The COP‐212 can retrieve 95.6% of gold even when gold was at very lower concentrations compared to other metals in the metal leachate.

## 4 Experimental Section

4.1

4.1.1

##### Materials

Meso‐tetraphenylporphyrin was purchased from Alfa Aesar. Gold (III) chloride trihydrate (HAuCl_4_·3H_2_O, ≥99.9%), copper chloride (CuCl_2_, 99.999%), and 5,10,15,20‐tetra(4‐pyridyl)‐21H,23H‐porphine were obtained from Merck. DCM (99.5%), chloroform (CHCl_3_, 99.5%), DCE (99.0%), methanol, hydrochloric acid (35.0–37.0%), nitric acid (68.0–70.0%), thiourea, silver nitrate (AgNO_3_, 99.8%), cobalt chloride hexahydrate (CoCl_2_·6H_2_O, 97.0%), and nickel chloride hexahydrate (NiCl_2_·6H_2_O, 97.0%) were purchased from Samchun. Aluminum (III) chloride anhydrous (AlCl_3_, 95%) was from Junsei. Potassium tetrachloroplatinate (II) (K_2_PtCl_4_, 46–47% Pt) and potassium tetrachloropalladate (II) (K_2_PdCl_4_, min 32.0% Pd) were purchased from Acros Organics. All the solvents were used without purification. For all metal adsorption and desorption experiments, deionized water (DIW) obtained from MiliQ (18.2 MQ cm at 25 °C) system was used.

##### Synthesis of COP‐210, 211, and 212

The COP‐210 was prepared by the following procedure. A 500 mg of meso‐tetraphenylporphyrin was added into 30 mL glass vial. Then, 672 mg of AlCl_3_, 10 mL of DCM, and stirring bar were added into the vial. The reaction mixture was heated to 40 °C, closed with cap, and stirred for 48 h (**caution**: HCl might build up pressure in the vial). After 48 h, the reaction was quenched by slowly adding 10 mL of methanol (**caution**: AlCl_3_ and methanol reaction is highly exothermic), then solid was filtered and washed with methanol and chloroform (10 mL each). Resultant solid was sonicated for 30 min and soaked in 6 m HCl (18% HCl in methanol) overnight. Then, product was washed in a Soxhlet extractor with 100 mL chloroform and 100 mL methanol for 24 h each. After washing, product was dried at 100 °C under vacuum for 12 h. Yield was 490 mg. For the synthesis of COP‐211 and COP‐212, 10 mL of chloroform and DCE were used, respectively, instead of DCM in the COP‐210 synthesis. The same procedure was used to prepare COP‐211 and COP‐212. Final products were 510 mg of COP‐211 and 570 mg of COP‐212.

##### Characterization of Pore Structures and Gas Adsorption Study

Porosity characterization of polymers was conducted from argon adsorption isotherms using a Micromeritics 3FLEX accelerated surface area and porosimetry analyzer at 87 K. Prior to measurement, samples were degassed at 423 K for 6 h under vacuum. The specific surface areas were derived from BET method. All pore size distributions were calculated by the Micromeritics 3FLEX software using an NLDFT model with slit pores. Adsorption and desorption of CO_2_ and CH_4_ were conducted at 273 K and 298 K, respectively, and H_2_ was at 77 K after degassing the samples prior to each measurement.

##### Single Metal Uptake Studies

Metal salts (HAuCl_4_·3H_2_O, K_2_PtCl_4_, K_2_PdCl_4_, AgNO_3_, and CuCl_2_) were dissolved separately in DIW to prepare 50 mL of 3000 ppm stock metal solutions, respectively. A 500 mg of COPs was placed in each metal solution. After shaking the mixtures at 8 rpm for 48 h, the COPs were separated by filtration and washed thoroughly with DIW. The metal adsorbed COPs were dried in air and then in a vacuum oven overnight at 100 °C.

##### Gold Adsorption Isotherms

From the gold stock solution, the gold solutions of 20, 100, 500, 1000, 2000, and 5000 ppm were prepared. After adding ≈10 mg of COPs to the solution at each concentration, the mixtures were shaken at 8 rpm for 48 h. The COPs were separated by using syringe filter units. The gold concentrations were measured by ICP–MS and the adsorption amount at each concentration was calculated as follows

(1) $$\text{Gold}\;\text{adsorption}\;\text{amount}\;(\text{Au}\;\text{mg/polymer}\;\text{mg})=\frac{Q_{i}–Q_{e}\left(\text{mg}\right)}{\text{Polymer}\;\left(\text{mg}\right)}$$

where *Q*_i_ is the gold amount in the initial solution and *Q*_e_ is that at equilibrium.

The gold adsorption isotherms were fitted by Langmuir adsorption model. The equation of Langmuir model is represented as follows

(2) $$Q_{e}\;=\;\frac{Q_{m\;}\cdot \;K_{L\;}\cdot \;C_{e}}{1\;+\;K_{L\;}\cdot \;C_{e}}$$

where *Q*_e_ (*g*_Au_/*g*_Ads_) is the quantity of adsorbed metal ions in a gram of adsorbent at equilibrium, *C*_e_ (mg L^−1^) is the equilibrium concentration, *Q*_m_ (*g*_Au_/*g*_Ads_) is the maximum uptake amounts of metal ions in a gram of adsorbent, and *K*_L_ is the Langmuir constant. *Q*_m_, *K*_L_, and *R* ^2^ were derived from the aforementioned equation and arranged in Table S3, Supporting Information.

##### Application of COP‐212 in Gold Recovery from e‐Waste

The PCBs were obtained from the local junk shop. The metals in PCBs were leached by following the modified method described in the literature.^[^ ^48^ ^]^ First, the PCBs were soaked in 10 m of NaOH solution for a day to remove the epoxy on the surface. The PCBs were taken out and washed with tap water. Then, the PCBs were soaked in 4 L of 1 m of HCl and HNO_3_ solution. The temperature of the solution was raised to 40 °C and held for 2 days. The PCBs were taken out and the acidic solution was filtered to remove any undissolved parts. KOH was added to the solution to reach a positive pH value and DIW was added to make a 5 L of the final solution. A 50 mg of COP‐212 was added to the 100 g of the solution and the mixture was stirred for 24 h. After filtering, COP‐212 was washed thoroughly with DIW. The loaded metals on COP‐212 were analyzed by ICP–MS after dissolving the polymer using microwave instrument. The metal amounts in the polymer dissolved solution were compared with the metal concentrations of the solution before COP‐212 addition.

## Conflict of Interest

The authors declare no conflict of interest.

## Data Availability Statement

Research data are not shared.

## Supporting information

Supplementary Material
