# Supplementary material for: Alkyl‐Linked Porphyrin Porous Polymers for Gas Capture and Precious Metal Adsorption
Source: Small Sci. 2021 May 5;1(6):2000078. doi: 10.1002/smsc.202000078 (PMC11935786; doi:10.1002/smsc.202000078)
Supplement: Supplementary file 1 — Supplementary Material [file SMSC-1-2000078-s001.pdf]

Supporting Information

Alkyl-linked porphyrin porous polymers for gas capture and precious metal adsorption

*Yeongran Hong, Vepa Rozyyev, Cafer T. Yavuz**

Y. H.
Department of Chemical and Biomolecular Engineering, Korea Advanced Institute of Science and Technology (KAIST), 291 Daehak-ro, Yuseong-gu, Daejeon, 34141, Republic of Korea

V. R.
Graduate School of EEWS, KAIST, 291 Daehak-ro, Yuseong-gu, Daejeon, 34141, Republic of Korea
Pritzker School of Molecular Engineering, the University of Chicago, 5640 South Ellis Avenue, Chicago, Illinois, 60637, USA.

Prof. C. T. Y.
Department of Chemical and Biomolecular Engineering, Korea Advanced Institute of Science and Technology (KAIST), 291 Daehak-ro, Yuseong-gu, Daejeon, 34141, Republic of Korea
Graduate School of EEWS, KAIST, 291 Daehak-ro, Yuseong-gu, Daejeon, 34141, Republic of Korea
Advanced Membranes and Porous Materials Center (AMPM), Physical Sciences and Engineering (PSE), King Abdullah University of Science and Technology (KAUST), Thuwal 23955-6900, Saudi Arabia
KAUST Catalysis Center (KCC), Physical Sciences and Engineering (PSE), King Abdullah University of Science and Technology (KAUST), Thuwal 23955-6900, Saudi Arabia

E-mail: yavuz@kaist.ac.kr, cafer.yavuz@kaust.edu.sa

1. Materials and methods

Materials

Meso-tetraphenylporphine was purchased from Alfa Aesar. Gold (III) chloride trihydrate ($HAuCl_4 \cdot 3H_2O$, ≥ 99.9 %), copper chloride ($CuCl_2$, 99.999 %) and 5,10,15,20-tetra(4-pyridyl)-21H,23H-porphine were obtained from Merck. Dichloromethane (DCM, 99.5 %), chloroform ($CHCl_3$, 99.5 %), 1,2-dichloroethane (DCE, 99.0%), methanol, hydrochloric acid (35.0-37.0 %), nitric acid (68.0-70.0 %), thiourea, silver nitrate ($AgNO_3$, 99.8 %), cobalt chloride hexahydrate ($CoCl_2 \cdot 6H_2O$, 97.0 %), and nickel chloride hexahydrate ($NiCl_2 \cdot 6H_2O$, 97.0 %) were purchased from Samchun. Aluminium (III) chloride anhydrous ($AlCl_3$, 95 %) was from Junsei. Potassium tetrachloroplatinate (II) (K_2PtCl_4, 46-47 % Pt) and potassium tetrachloropalladate (II) (K_2PdCl_4, min 32.0 % Pd) were purchased from Acros Organics. All the solvents were used without purification. For all metal adsorption and desorption experiments, deionized water (DIW) obtained from MiliQ (18.2 MΩ·cm at 25 ^oC) system was used.

Characterization

Fourier transform attenuated total reflectance-infra-red spectra (FT-ATR-IR) was recorded with a Shimadzu IRTracer, Gladi-ATR 10 model Fourier transform infrared spectrometer. The background data was deleted from the sample data; baseline was corrected, and data was smoothed before recovery. Elemental analysis for C, H, N, and O was carried out using a FLASH 2000 series of Thermo Scientific. Porosity and gas adsorption-desorption characterization of polymers were carried out from argon adsorption isotherms using a Micromeritics 3FLEX accelerated surface area and porosimetry analyzer at 87 K. Prior to measurement, samples were degassed at 423 K for 6 hours under vacuum. The specific surface areas were derived from Brunauer-Emmett-Teller (BET) method. All pore size distributions were

calculated by the Micromeritics 3FLEX software using an NLDFT model with slit pores. Powder X-ray diffraction (PXRD) patterns of samples were measured over the 2θ range of 20-90° on a Rigaku D/MAX-2500 (18 kW) multi-purpose high power X-ray diffractometer. X-ray photoelectron spectroscopy (XPS) was conducted on a K-alpha model of Thermo VG Scientific equipped with a microfocused monochromator X-Ray source with the energy resolution of 0.5 eV full-width at half-maximum under ultrahigh vacuum condition of 10^{-9} Torr. Field emission transmission electron microscopy (TEM) images were recorded on a Talos F200X model of FEI. The ICP-MS instrument of Agilent 7700x model was used for the metal analysis. The multi-element standard solutions from Agilent technologies (Agilent part no. 8500-6940 and 8500-6948) were used for calibration and the metal selectivity tests. Thermogravimetric analysis (TGA) was carried out with a differential thermal gravimetry (DTG)-60A of Shimadzu at a heating rate of 10 ^{o}C min^{-1} up to 800 ^{o}C under air and nitrogen atmosphere, respectively. The ultraviolet–visible (UV/vis) absorbance spectra were obtained by Lambda 1050 from Perkin Elmer.

Metal selectivity

The ICP metal standard solutions were diluted to 100 ppb with the addition of DIW. 10 mL of three solutions were used as experimental groups and 10 mL of two solutions were used as control groups. In the 10 mL of three experimental solutions, approximately 10 mg of COP was added. Both experimental and control groups were shaken at 8 rpm for 24 hours and then filtered by using syringe filter units. The metal concentrations in the solutions were measured by ICP-MS. The metal adsorption efficiency of each metal was calculated according to the equation below:

$$Metal\ adsorption\ efficiency\ (\%) = \frac{C_c - C_e}{C_c}\ x\ 100\ \%$$

where C_c is the average metal concentration in the control group and C_e is that in the experimental group.

Gold adsorption at varying pH

100 mL of 1 ppm gold solutions were prepared from the gold stock solution. The pH of the solution was adjusted to 2, 4, 7, and 9 by adding a few drops of 0.1 M HCl and 0.1 M NaOH solutions. 30 mg of COPs was added to each solution, and the mixtures were magnetically stirred. After 0.5, 1, 3, 6, 18, and 24 hours, small portions of solutions (1 mL) were taken and filtered by using syringe filter units. The gold concentrations in each solution were measured by ICP-MS.

UV/Vis absorption measurement

Solution (1) was prepared as follow: 6 mg of 5,10,15,20-tetra(4-pyridyl)-21H,23H-porphine was dissolved in 1 L of aqueous 2 M HCl solution. In a 10 mL of vial, 5 mL of DIW and 5 mL of prepared porphyrin solution were added and mixed. Solution (2) was made as follow: Metal stock solutions were prepared by dissolving metal salts in DIW. The metal concentrations were confirmed by ICP-MS and then, controlled to 5 mM by adding more water. In a 10 mL of vial, 5 mL of aqueous 2 M HCl solution and 5 mL of prepared 5 mM metal solution were mixed. Solution (3) was prepared as follow: 5 mL of porphyrin solution in Solution (1) and 5 mL of 1000 ppm metal solution made in Solution (2) were added to a 10 mL of vial and mixed. Solution (1), (2), and (3) were shaken at 8 rpm and room temperature. After 24 hours, UV/vis spectra of three solutions for each metal were measured.

Metal desorption

10 mg of metal loaded COPs was placed in the 10 mL of vials. Desorption solutions were added to the vials and the vials were placed in the oil bath to maintain the temperature at 40 ^{o}C. The mixtures were magnetically stirred and after 24 hours, the COPs were separated using syringe

filter units. The metal concentrations in filtrates were measured by ICP-MS. The metal amounts were calculated from the filtrate volumes and the values were compared with the initial metal adsorption amounts to yield the desorption efficiencies.

Gold adsorption-desorption cycles

300 mg of COPs was placed in the 50 mL of gold solutions at 3000 ppm. The mixtures were stirred for 24 hours, filtered and washed with DIW. The Au-COPs were dried in air and in vacuum at 100 ^{o}C overnight. 10 mg of Au-COPs was added to the HCl/HNO_3 mixtures (conc. HCl : conc. HNO_3 = 3:7). The Au-COPs in the acid mixtures were dissolved by microwave instrument. The gold concentrations were measured by ICP-MS and the adsorbed gold amounts in a gram of polymer were calculated.

The Au-COPs were placed in the 100 mL mixture of 0.1M $SC(NH_2)_2$, 1 M HCl, and 1 M HNO_3 . The mixtures were stirred at 40 ^{o}C for 28 hours. The COPs were filtered and washed with DIW. The gold concentrations in filtrate were measured by ICP-MS and compared with the gold adsorption amounts to yield the desorption efficiencies. The adsorption and desorption processes were repeated three times.

2. Supporting figures

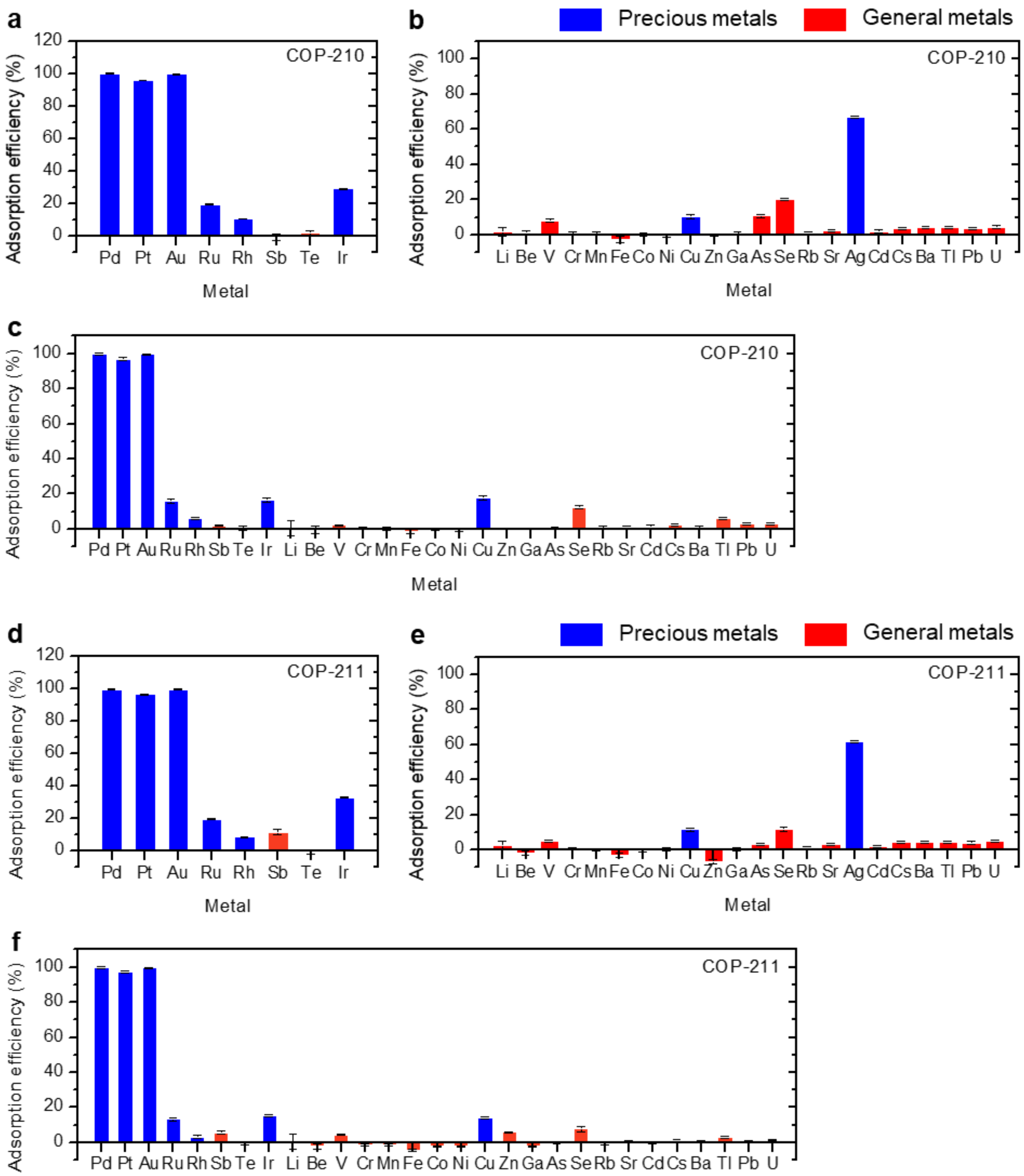

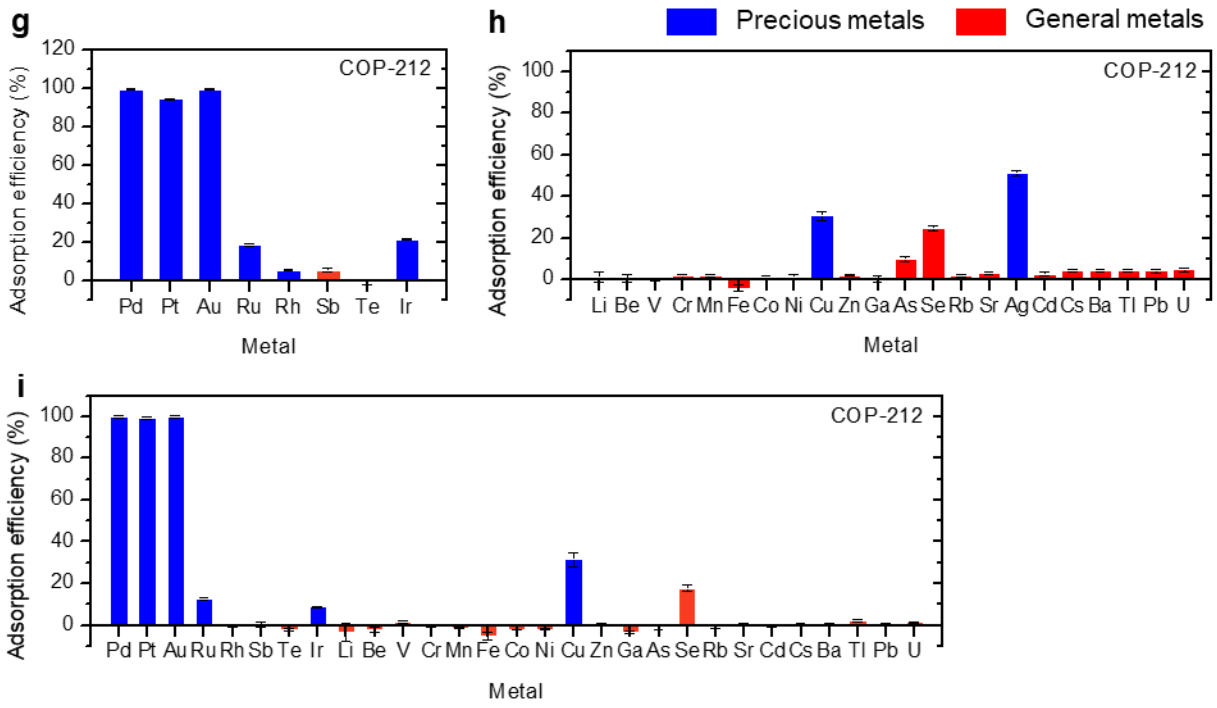

Figure S1. Metal selectivity test results. COP-210 tested in (a) the standard solution-1, (b) the standard solution-2, and (c) mixed standard solution-1 and -2. COP-211 tested in (d) the standard solution-1, (e) the standard solution-2, and (f) mixed standard solution-1 and -2. COP-212 tested in (g) the standard solution-1, (h) the standard solution-2, and (i) mixed standard solution-1 and -2.

* The negative adsorption efficiencies were observed in several elements such as sodium, magnesium, aluminium, potassium, and calcium. These metals are commonly found in water and on the experimental tools. Hafnium was unstable in this experimental condition, showing increased concentrations after treatment. These elements were excluded for the clarity of the figures. Silver was also not included in the Figure S1a-c, since insoluble silver chloride is possibly formed with HCl, which is the matrix component of standard solution-1, when the two standard solutions were mixed.

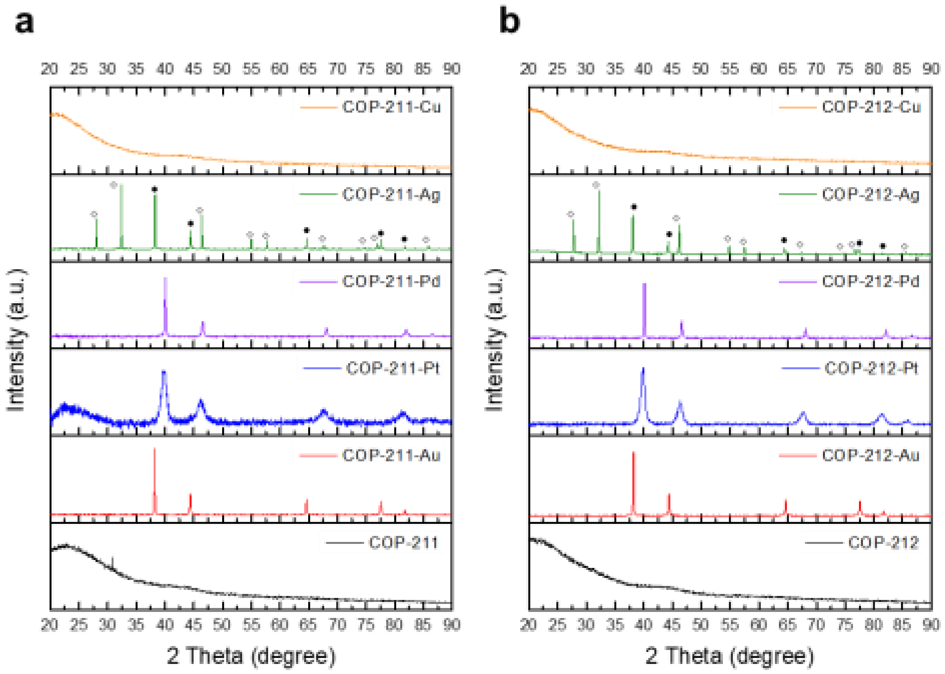

Figure S2. XRD patterns of copper, silver, palladium, platinum, and gold adsorbed (a) COP-211 and (b) COP-212 compared to the COP-211 and COP-212 without metal loading. In the XRD patterns of COP-211-Ag and COP-212-Ag, the symbols of diamond (◇) and filled circle (●) indicate silver chloride and silver nanoparticles, respectively. The ICDD numbers of gold, platinum, palladium, silver particles and silver chloride are 00-004-0784, 00-004-0802, 00-005-0681, 03-065-2871, and 00-031-1238, respectively.

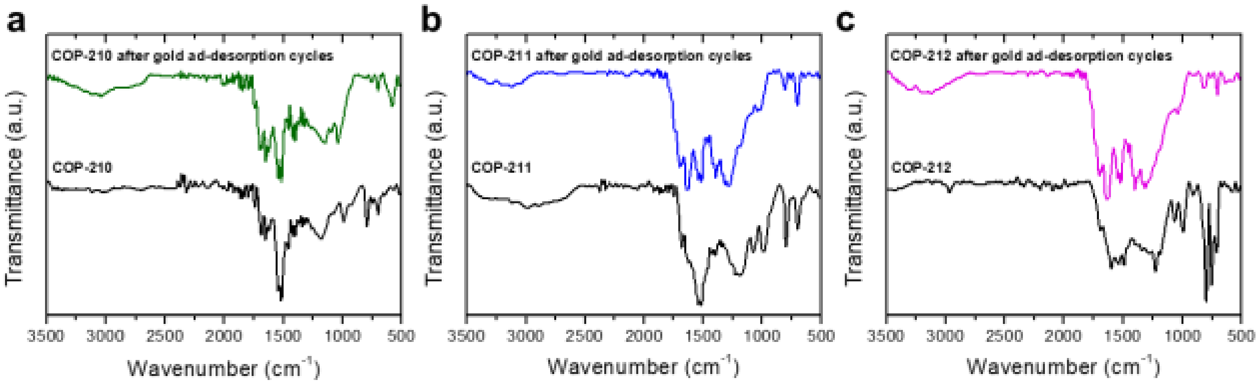

Figure S3. FT-IR spectra of (a) COP-210, (b) COP-211, and (c) COP-212 after three cycles of gold adsorption and desorption compared to pristine COPs.

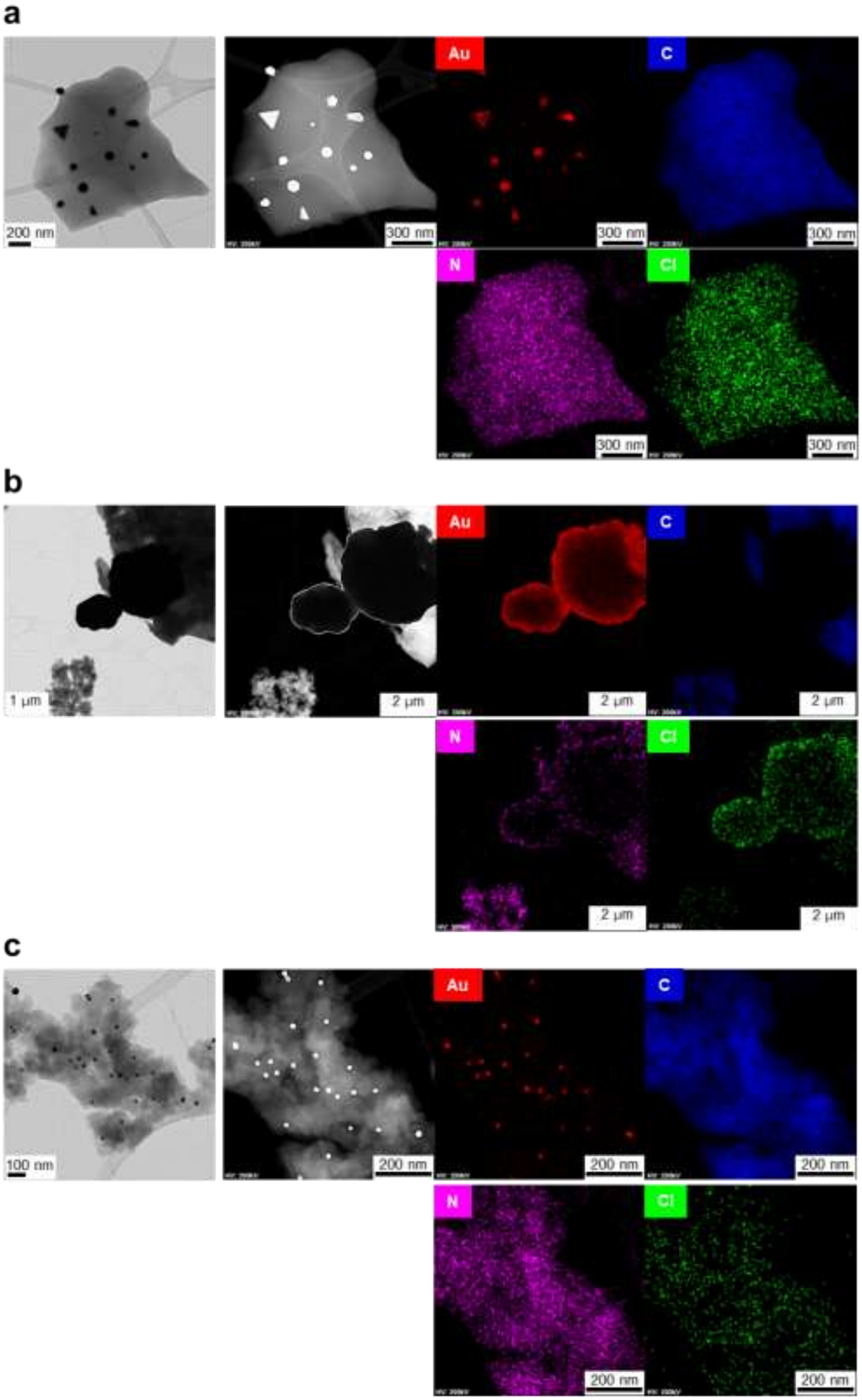

Figure S4. TEM and STEM images of gold loaded (a) COP-210, (b) COP-211, and (c) COP-212.

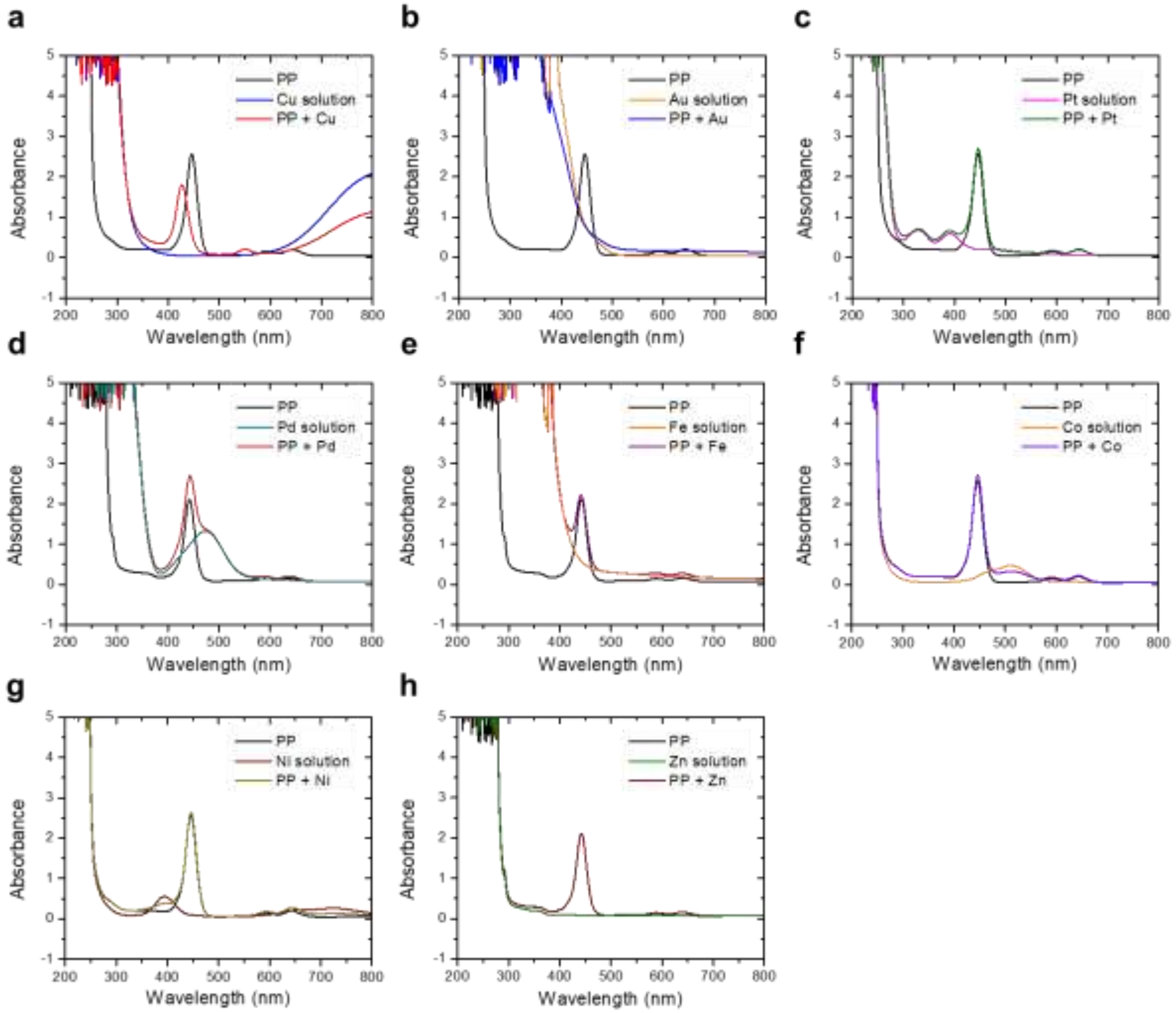

Figure S5. UV/Vis absorption changes after addition of porphyrin solution to (a) copper, (b) gold, (c) platinum, (d) palladium, (e) iron, (f) cobalt, (g) nickel, and (h) zinc solutions.

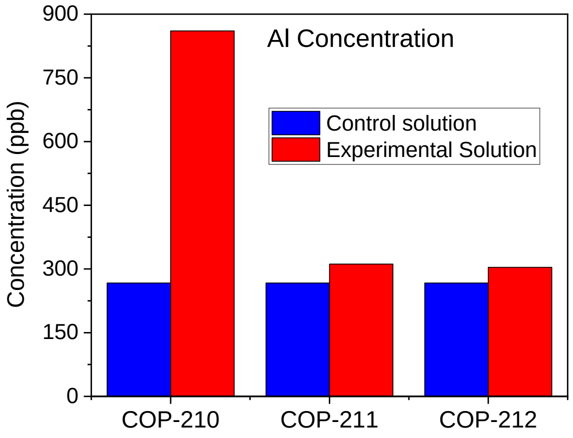

Figure S6. ICP-MS aluminum concentration before and after treated with COP-210, COP-211, and COP-212.

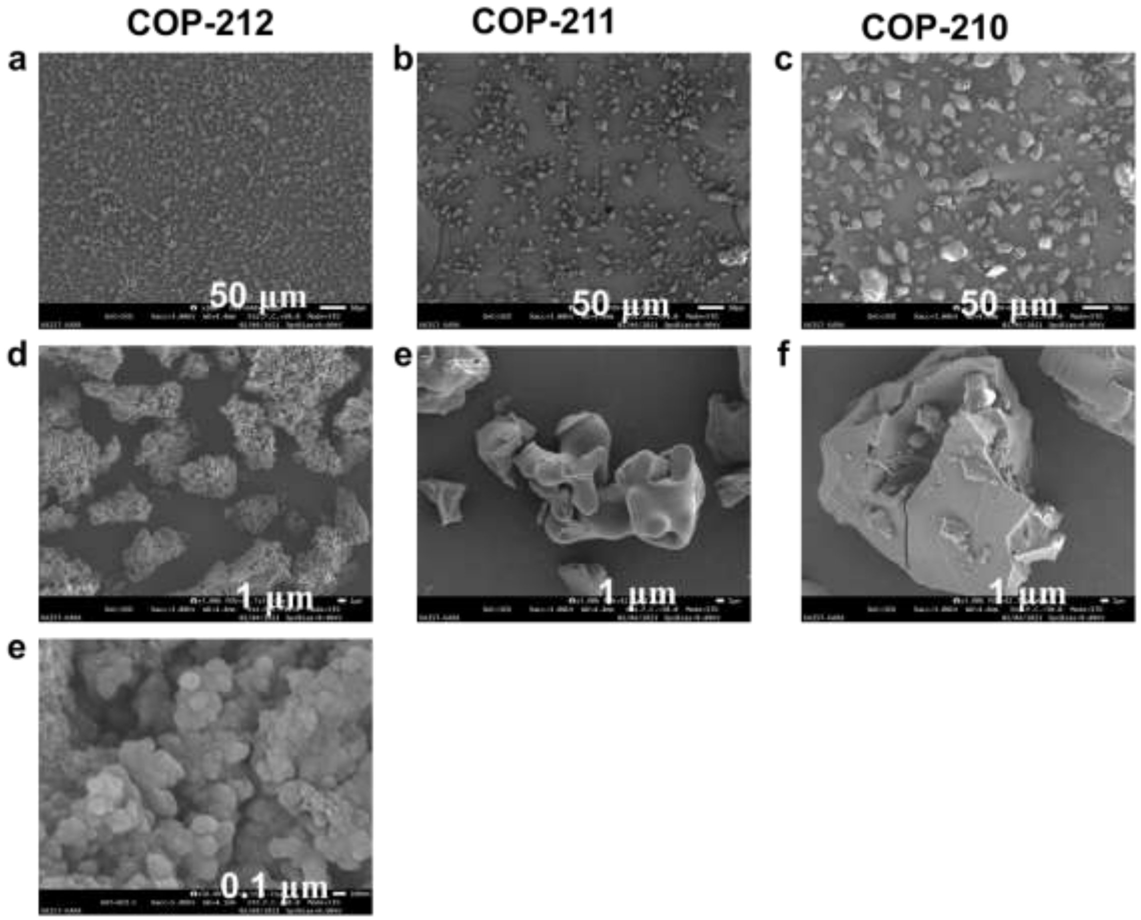

Figure S7. Scanning electron microscopy (SEM) images of COP-210, COP-211, and COP-212

3. Supporting tables

Table S1. Elemental composition (%) of (a) COP-210, COP-211, and COP-212 and (b) metal loaded COP-210, COP-211, and COP-212.

(a)

Sample	C (%)	N (%)	H (%)	Al* (%)	C at. Ratio (Theoretical)	N at. Ratio (Theoretical)	H at. Ratio (Theoretical)
COP-210	73.58	6.86	4.11	4.29	50.0 (46)	4 (4)	33.55 (30)
COP-211	68.25	6.31	3.65	0.89	50.4 (45.33)	4 (4)	32.13 (27.33)
COP-212	73.70	5.96	4.39	1.54	57.7 (48)	4 (4)	41.25 (34)
Meso-Tetraphenyl porphine	85.97	9.11	4.92	0	44	4	30

*Al content was measured from leftover mass (Al_2O_3) after air TGA measurement.

(b)

Sample	C	N	H	O	Metal
COP-210-Au	41.37	3.30	3.11	9.40	30.61
COP-211-Au	43.28	4.01	2.47	8.66	31.73
COP-212-Au	50.27	4.14	3.21	7.28	30.03
COP-210-Pt	58.48	4.90	3.46	13.11	8.50
COP-211-Pt	59.50	5.39	3.43	10.47	9.34
COP-212-Pt	62.93	5.05	3.84	10.50	12.06
COP-210-Pd	59.71	4.59	3.27	9.83	11.37
COP-211-Pd	58.79	4.75	2.97	9.75	10.88
COP-212-Pd	65.38	4.96	3.59	7.94	9.77
COP-210-Ag	60.11	4.81	3.26	9.70	8.92
COP-211-Ag	60.71	5.41	3.34	12.06	8.65
COP-212-Ag	66.17	5.19	3.91	9.19	8.89
COP-210-Cu	68.40	5.88	3.92	10.78	2.93
COP-211-Cu	68.15	6.22	3.78	10.59	1.37
COP-212-Cu	72.45	5.94	4.25	8.40	2.17

Table S2. (a) Desorption efficiencies of metal loaded COPs at different conditions and (b) gold adsorption and desorption amounts, and desorption efficiencies at three ad/desorption processes of COP-210, COP-211, and COP-212.

(a)

Desorption conditions	0.1 M $SC(NH_2)_2$ + 1 M HCl + 1 M HNO_3 (40 ^oC, 24 h)
COP-210-Au	92.15
COP-211-Au	87.67
COP-212-Au	99.98
COP-210-Pt	24.73
COP-211-Pt	35.25
COP-212-Pt	23.4
COP-210-Pd	31.8
COP-211-Pd	34.04
COP-212-Pd	33.62
COP-210-Cu	28.89
COP-211-Cu	21.50
COP-212-Cu	20.81
Desorption conditions	0.1 M $SC(NH_2)_2$ + 1 M HNO_3 (40 ^oC, 24 h)
COP-210-Ag	100
COP-211-Ag	100
COP-212-Ag	100

(b)

Sample	Cycle number	Adsorption amounts (%)	Desorption amounts (%)	Desorption efficiency (%)
COP-210	1	30.61	29.12	90.6
	2	38.57	34.71	89.99
	3	50.28	37.93	75.44
COP-211	1	31.73	28.75	95.14
	2	36.89	32.21	87.31
	3	47.62	43.29	90.91
COP-212	1	30.03	29.78	99.18
	2	42.01	29.85	71.05
	3	49.03	32.54	66.37

Table S3. Information on the gold adsorption Langmuir isotherms of COP-210, COP-211, and COP-212.

	Gold adsorption capacity (g g^{-1})	R^2	Langmuir constant (K_L, L mg^{-1})
COP-210	1.176	0.965	0.00195
COP-211	0.901	0.925	0.0029
COP-212	1.250	0.989	0.00158

Table S4. Metals found in e-waste leaching solution and their concentrations and recovery efficiencies.

No.	Element	Amount (mg)	Recovery efficiency (%)
1	Li	0.576056	3.081462
2	Be	0.181654	0.00777
3	Al	53.17235	1.091188
4	Mn	0.547514	0.038306
5	Fe	23.30696	0.385854
6	Co	0.154849	0.020668
7	Ni	18.2054	0.0085
8	Cu	300.2124	0.769436
9	Zn	183.1189	0.005039
10	Rb	0.276527	0.011937
11	Sr	0.157433	0.032952
12	Pb	44.31674	0.00099
13	Sn	10.30994	2.239779
14	Au	0.389511	95.59328

Table S5. Metal adsorption performances comparison with other reported adsorbents.

No.	Adsorbent	Gold adsorption Capacity (g_{Au}/g)	Tested metals for selectivity	Gold adsorption Kinetics	Gold desorption and reusability	Reference
1	COP-210	1.176	31 metals	30 min	3 cycles	This study
2	COP-211	0.901	31 metals	30 min	3 cycles	This study
3	COP-212	1.250	31 metals	1 h	3 cycles	This study
4	Thiourea-modified polyethylenimine copolymer	3.152	ND	2 h	ND	Chen et al.[1]
5	Crosslinked persimmon tannin gel	1.52	Au(III), Pd(II), Pt(IV), Cu(II), Fe(III), Ni(II), Zn(II)	More than 30 h at 293 K	ND	Inoue et al.[2]
6	Cross-linked polysaccharide gels	1.491	Au, Pt, Pd, Fe, Cu	40 h at 293 K	ND	Inoue et al.[3]
7	Poly(Cys-g-Sty)	Au(III)-1.345, Pt(IV)-0.701, Pd(II)-0.442	Au(III), Pt(IV), Pd(II), Co(II), Ni(II), Zn(II), Mn(II)	18 h for Au, 1 min for Pt and Pd	99 % in 1st cycle and 68 % in 2nd cycle	Endo et al.[4]
8	BTU-PT gel	Au(III)-1.02, Pd(II)-0.192, Pt(IV)-0.131	Au(III), Pd(II), Pt(IV), Cu(II), Fe(III), Ni(II), Zn(II)	6 h for Au(III), 12 h for Pd(II) and Pt(IV)	5 cycles	Inoue et al.[5]
9	PE/PP-g-PDMAEMA	0.9493	Au(III), Cu(II), Fe(III), Ni(II), Pb(II)	96 % within 1 h	5 cycles	Li et al.[6]
10	Fe-BTC/PpPDA	0.934	Au, Cu, Ni, Ca, Mg, K, Na	2 min	3 regeneration cycles	Queen et al.[7]
11	BT-SiO_2	0.642 g/g at 323 K	Au, Pb, Ni, Cu, Zn	30 min	73 %	Shi et al.[8]
12	UiO-66-NH_2	Au(III)-0.495, Pt(IV)-0.193, Pd(II)-0.167	Co(II), Ni(II), Cu(II), Zn(II)	3 h for 100 ppm Au(III), Pt(IV), Pd(II)	5 cycles	Yun et al.[9]
13	UiO-66	Au(III)-0.280, Pt(IV)-0.166, Pd(II)-0.120	Co(II), Ni(II), Cu(II), Zn(II)	25 min for 100 ppm Au(III), Pt(IV), Pd(II)	5 cycles	Yun et al.[9]
14	COP-122-ao	0.4567	15 common metals	10 min	ND	Yavuz et al.[10]

15	3D bioMOF	598 mg of $AuCl_3$ / 1 g of adsorbent (0.389 g/g)	Au, Pd, Ni, Cu, Zn, Al	30 min	ND	Pardo et al.[11]
16	NH_2-MCM-41	0.275	Au, Cu, Fe, Pd, Pt	N/A	5 cycles	Yeung et al.[12]
17	SH-MCM-41	0.195	Au, Cu, Fe, Pd, Pt	N/A	5 cycles	Yeung et al.[12]
18	DTGA-XAD-16	0.035	Au, Ni, Cu, Sn, Fe, Cr, Se, Zn, Pb, Ba, As, Y	3 h	4 cycles	Kumar et al.[13]
19	MNP-G3	Pd(IV)-0.00362, Au(III)-0.00360, Pd(II)-0.00275, Ag(I)-0.00284	Au(III), Pd(II), Pd(IV), Ag(I), Zn(II)	90 % of Au within 8 h	6 cycles of Pd(IV) desorption	Lien et al.[14]
20	Imi-SBA-15	Pt-0.0178, Pd-0.00968	Pt, Pd, Cu, Ni, Cd	Pt and Pd adsorption within 6 h	Pt-71.45%, Pd-60.32%	Yi et al.[15]

Table S6. Gas adsorption performance comparisons of COPs with other reported adsorbents.

Adsorbent	SA_{BET} (m^2 g^{-1})	Chemistry	CO_2 uptake at 273K, 1.1 bar (mmol g^{-1})	CH_4 uptake at 273K, 1.1 bar (mmol g^{-1})	H_2 uptake at 77K, 1.1 bar (mmol g^{-1})	Reference
COP-210	856	Porphyrin, alkyl	4.3	1.33	8.88	This work
COP-211	790	Porphyrin, alkyl	3.47	1.44	8.24	This work
COP-212	685	Porphyrin, alkyl	3.01	1.30	7.11	This work
BILP-2	708	Benzimidazole	3.32	0.87	6.45	El-Kaderi et al.[16]
BILP-6	1261	Benzimidazole	4.79	1.68	10.9	El-Kaderi et al.[16]
CuPor-BDPC	442	Porphyrin, imine	1.25	0.20	1.98	Echegoyen et al.[17]
PAF-1	5600	Aromatic	2.05	1.25	7.5	Ben et al.[18]
PIM-1	740	Nitrile, aromatic ether	2.53	0.82	4.71	Song et al.[19]
TATHCP	997	Alkyl carbazole, aromatic	2.85	0.97	6.45	Sadak et al.[20]
COF-1	750	Boronate, aromatic	1.18	ND	5.36	Furukawa et al.[21]
COF-102	3620	Boronate, aromatic	0.84	0.94	6.25	Furukawa et al.[21]
BPL carbon	1250	Carbon	1.77	ND	7.82	Furukawa et al.[21]
PECONF-3	851	Phosphazene, aromatic	3.3	0.6	ND	Mohanty et al.[22]

References

[1] Y. Li, H. Tian, C. Xiao, J. Ding, X. Chen, *Green Chem.* **2014**, *16*, 4875-4878.
[2] M. Gurung, B. B. Adhikari, H. Kawakita, K. Ohto, K. Inoue, S. Alam, *Chem. Eng. J.* **2011**, *174*, 556-563.
[3] B. Pangeni, H. Paudyal, M. Abe, K. Inoue, H. Kawakita, K. Ohto, B. B. Adhikari, S. Alam, *Green Chem.* **2012**, *14*, 1917-1927.
[4] H. Akbulut, S. Yamada, T. Endo, *RSC Adv.* **2016**, *6*, 108689-108696.
[5] M. Gurung, B. B. Adhikari, H. Kawakita, K. Ohto, K. Inoue, S. Alam, *Ind. Eng. Chem. Res.* **2012**, *51*, 11901-11913.
[6] X. Liu, J. Ao, X. Yang, C. Ling, B. Zhang, Z. Wang, M. Yu, R. Shen, H. Ma, J. Li, *J. Appl. Polym. Sci.* **2017**, *134*.
[7] D. T. Sun, N. Gasilova, S. Yang, E. Oveisi, W. L. Queen, *J. Am. Chem. Soc.* **2018**, *140*, 16697-16703.
[8] X. Huang, Y. Wang, X. Liao, B. Shi, *J. Hazard. Mater.* **2010**, *183*, 793-798.
[9] S. Lin, D. H. K. Reddy, J. K. Bediako, M.-H. Song, W. Wei, J.-A. Kim, Y.-S. Yun, *J. Mater. Chem. A* **2017**, *5*, 13557-13564.
[10] N. A. Dogan, Y. Hong, E. Ozdemir, C. T. Yavuz, *ACS Sustain. Chem. Eng.* **2018**, *7*, 123-128.
[11] M. Mon, J. s. Ferrando-Soria, T. Grancha, F. R. Fortea-Pérez, J. Gascon, A. Leyva-Pérez, D. Armentano, E. Pardo, *J. Am. Chem. Soc.* **2016**, *138*, 7864-7867.
[12] K. F. Lam, C. M. Fong, K. L. Yeung, G. Mckay, *Chem. Eng. J.* **2008**, *145*, 185-195.
[13] A. B. Kanagare, K. Singh, M. Kumar, M. Yadav, R. Ruhela, A. Singh, A. Kumar, V. Shinde, *Ind. Eng. Chem. Res.* **2016**, *55*, 12644-12654.
[14] C.-H. Yen, H.-L. Lien, J.-S. Chung, H.-D. Yeh, *J. Hazard. Mater.* **2017**, *322*, 215-222.
[15] T. Kang, Y. Park, K. Choi, J. S. Lee, J. Yi, *J. Mater. Chem.* **2004**, *14*, 1043-1049.
[16] M. G. Rabbani, H. M. El-Kaderi, *Chem. Mater.* **2012**, *24*, 1511-1517.
[17] V. S. P. K. Neti, X. Wu, S. Deng, L. Echegoyen, *Polym. Chem.* **2013**, *4*, 4566-4569.
[18] T. Ben, C. Pei, D. Zhang, J. Xu, F. Deng, X. Jing, S. Qiu, *Energy Environ. Sci.* **2011**, *4*, 3991-3999.
[19] Q. Song, S. Cao, R. H. Pritchard, B. Ghalei, S. A. Al-Muhtaseb, E. M. Terentjev, A. K. Cheetham, E. Sivaniah, *Nat. Commun.* **2014**, *5*, 1-12.
[20] A. E. Sadak, E. Karakus, Y. M. Chumakov, N. A. Dogan, C. T. Yavuz, *ACS Appl. Energy Mater.* **2020**, *3*, 4983-4994.
[21] H. Furukawa, O. M. Yaghi, *J. Am. Chem. Soc.* **2009**, *131*, 8875-8883.
[22] P. Mohanty, L. D. Kull, K. Landskron, *Nat. Commun.* **2011**, *2*, 1-6.
